# Effectiveness of an Intervention to Prevent Ultra‐Processed Foods and Added Sugar in the First Year of Life: A Multicentre Randomised Controlled Trial in Brazil

**DOI:** 10.1111/jhn.70022

**Published:** 2025-02-17

**Authors:** Paola S. Baratto, Daniel J. Hoffman, Júlia L. Valmórbida, Paula S. Leffa, Carlos A. Feldens, Márcia R. Vitolo

**Affiliations:** ^1^ Graduate Program in Pediatrics, Child and Adolescent Health Federal University of Health Sciences of Porto Alegre Porto Alegre Rio Grande do Sul Brazil; ^2^ Department of Nutritional Sciences New Jersey Institute for Food, Nutrition, and Health Rutgers, The State University of New Jersey New Brunswick New Jersey USA; ^3^ Graduate Program in Health Sciences Federal University of Health Sciences of Porto Alegre Porto Alegre Rio Grande do Sul Brazil; ^4^ Department of Preventive and Social Dentistry Federal University of Rio Grande do Sul, Porto Alegre, Brazil Porto Alegre Brazil

**Keywords:** dietary sugars, early intervention, infant and child feeding, nutrition education intervention, ultra‐processed foods

## Abstract

**Background:**

The early consumption of ultra‐processed foods (UPFs) and added sugars (AS) has been linked to adverse outcomes in infancy. The objective of this study was to determine the effectiveness of a dietary counselling strategy to prevent the consumption of UPFs and AS in the first year of life.

**Methodology:**

A multicentre randomised controlled trial was conducted with 516 mother–child pairs in three state capitals of Brazil. Mothers were randomly assigned to the control group (CG) or intervention group (IG) after childbirth. The IG received orientation based on UNICEF dietary guidelines and five monthly telephone calls to reinforce the intervention. Dietary intake was measured using food introduction questionnaires and 24‐h recalls during home visits at 6 and 12 months. Between‐group differences were analysed by generalised estimating equations and presented as mean difference (95% CI).

**Results:**

Children in the IG had lower UPF intake at 6 and 12 months of age (−20.69 g/day; 95% CI: −37.87 to −3.50; *p* = 0.018 and −32.51 g/day; 95% CI: −61.03 to −3.99; *p* = 0.025) and lower AS intake at 12 months of age (−4.92 g/day; 95% CI: −9.43 to −0.41; *p* = 0.033). The intervention also had a positive impact on the period of exclusive breastfeeding, reducing the offer of infant formula, cow's milk, and toddler milk in the first year of life.

**Principal Conclusions:**

The dietary counselling strategy was effective at preventing the early consumption of UPFs and AS in the first year of life. Future research should focus on social and cultural barriers to improve adherence to infant feeding interventions.

## Summary

- Counselling mothers and family members about the health risks of AS and UPFs in a postnatal environment was effective in preventing the early offer and reducing the consumption of AS and UPFs in the first year of life.
- The emphasis on preventing nonrecommended foods enabled mothers in the intervention group to breastfeed exclusively for a longer period of time, preventing the culturally encouraged early offer of tea, breast milk substitutes, juices and homemade porridges.

## 1 Introduction

The consumption of ultra‐processed foods (UPFs) (i.e., highly processed formulations of ingredients that result from a set of industrial processes) has increased across all ages throughout the world [1, 2]. In infancy, UPFs are offered as part of complementary feeding [3], comprising up to 44% of total daily energy intake in the first year of life [4] and from 43.7% to 67% in older children [5, 6, 7, 8, 9]. Moreover, the consumption of added sugars (AS) has increased rapidly from birth to 12 months of age and gradually from 2 years of age onwards [10]. Therefore, it is of great public health importance to determine how to prevent the early introduction of UPFs and AS to prevent metabolic diseases in children and adults.

Children who consume high amounts of UPFs are more likely to have excessive body fat [11], asthma, food addictions [12], unhealthy glycaemic and lipid profiles [13, 14, 15] and high blood pressure [16]. AS consumption can lead to cardiovascular disease, type 2 diabetes [17] and dental caries [18, 19] and can reinforce the innate preference for sweetened foods and beverages [20]. The American Heart Association recommends avoiding AS in the first 2 years of life [17] and choosing minimally processed foods over UPFs [21]. It is, therefore, important to develop effective programmes for the prevention of the early consumption of UPFs and AS among children.

Numerous intervention studies have attempted to promote breastfeeding and healthy complementary feeding practices but have not focused on the avoidance of nonrecommended foods [22]. Most randomised trials on reducing the consumption of nonrecommended foods target school‐aged children [23, 24, 25] rather than infants and/or focus only on sugar‐sweetened beverages [26, 27, 28] rather than UPFs. Even after careful research, we found only four randomised studies that sought to educate mothers on the importance of improving infant feeding practices. In the first of these studies, the intervention was specifically tailored to Native American mothers [29], which limits the generalisability of the results. Another study involved several home visits in the first 12 months of age [30], limiting reproducibility. In the third, most information on child nutrition was collected at the 6‐month assessment, when the introduction of food had already begun [31]. Finally, the last intervention targeted teenage mothers, who may respond to nutrition education interventions differently than the general population [32].

Therefore, we developed a multicentre initiative involving a dietary counselling strategy for mothers and family members at maternity hospitals shortly after the birth of their children focusing on preventing the offer of UPFs and AS. We hypothesised that early dietary counselling to mothers and family members can prevent the early offer of nonrecommended foods, such as AS and UPFs.

## 2 Methods

### 2.1 Trial Design

**Figure 1:**
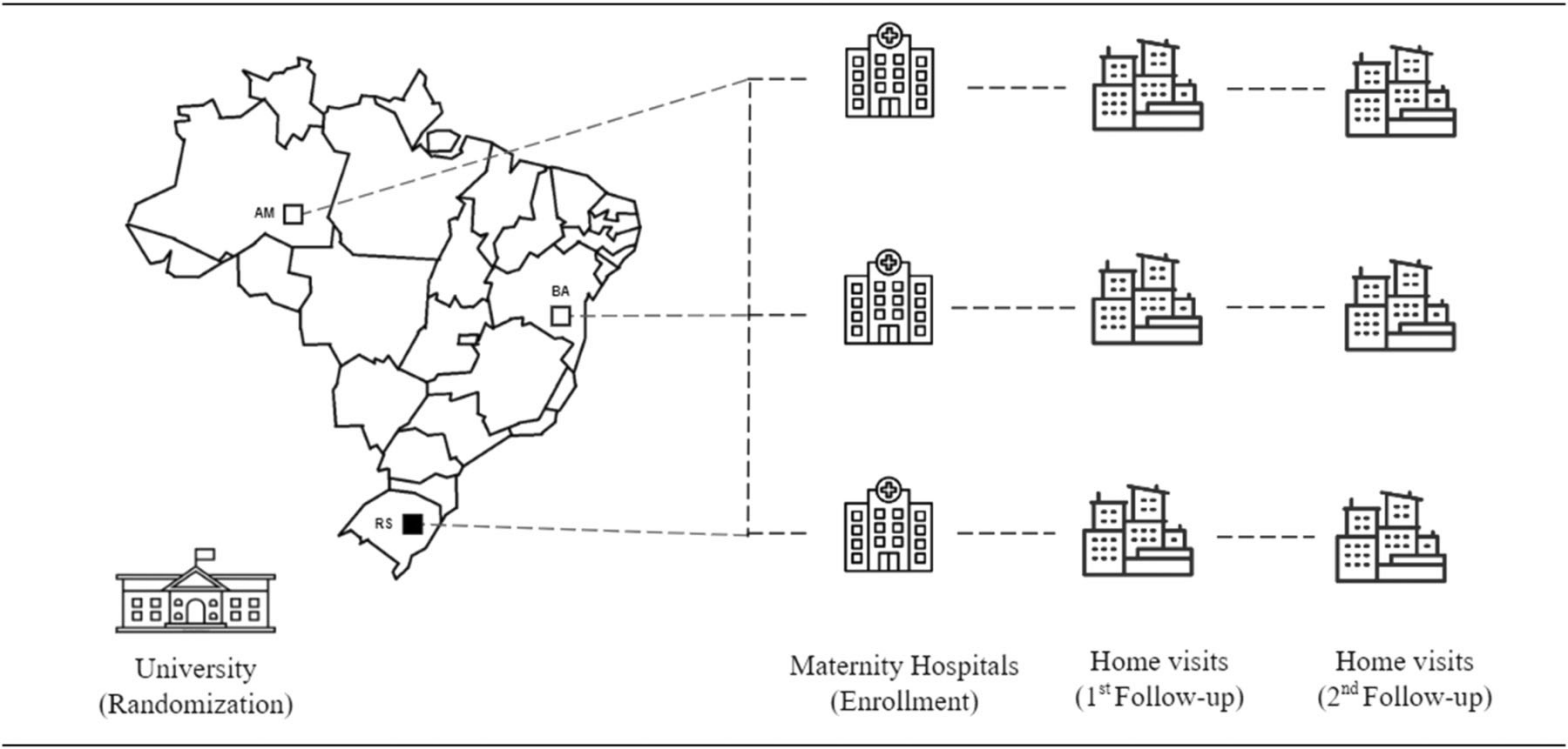
Study location (Brazil). Enrolment (newborn children) = from September to December 2018; 1st follow‐up (at 6 months of age) = from April to August 2019; 2nd follow‐up (at 12 months of age) = from September 2019 to January 2020.

A multicentre randomised controlled trial was carried out with mothers and newborns at maternity wards in three state capitals of Brazil (Manaus, Salvador and Porto Alegre). All hospitals were part of Brazil's national health system, which provides free access to healthcare predominantly to low‐income families. Follow‐up was conducted through home visits when the children were 6 and 12 months of age (Figure 1). These state capitals were chosen to represent different socioeconomic backgrounds, as Manaus is located in the northern region of Brazil, which comprises most of the Amazon rainforest, and Salvador and Porto Alegre are, respectively, situated in the northeastern and southern regions. According to the World Bank classification, Brazil is an upper middle‐income country with over 200 million inhabitants, a Human Capital Index of 0.6 (scale from 0 to 1) and an under‐five mortality rate of 15 per 1000 live births (https://data.worldbank.org/).

The random allocation sequence was created at the university in the city of Porto Alegre by a single research staff member not directly involved in participant enrolment using the Sealed Envelope® software programme (Sealed Envelope Ltd., https://sealedenvelope.com/). The randomisation scheme involved 4‐week blocks (i.e., intervention, control, intervention, and control) for each centre at the beginning of each month of data collection to avoid the imbalance in sample size between groups and avoid ‘contamination’ among the participants. All mothers were allocated to the intervention group or control group and enroled in the study by the interviewers based on the child's week of birth until the sample was completed. Women who gave birth in the weeks allocated to the intervention group received the standard hospital routine in addition to a single dietary counselling session, two booklets and five monthly phone calls to reinforce the intervention. Women who gave birth in the weeks allocated to the control group received the standard hospital routine. The final sample had a 1:1 ratio between groups.

This study received approval from the Ethics Committee of the Federal University of Health Sciences of Porto Alegre and was registered in clinicaltrials.gov (n° NCT03841123). All mothers provided written informed assent on behalf of their newborns. The procedures were performed in accordance with Resolution 466/12 of the Brazilian National Board of Health and the Declaration of Helsinki. To improve the quality of this report, we followed the Consolidated Standards of Reporting Trials (Table S1) [33].

### 2.2 Participants

Enrolment took place at three selected maternity wards designated by the Baby‐Friendly Hospital Initiative (BFHI) [34] and nationally recognised to support the Ten Steps to Successful Breastfeeding. To obtain the sample size within the stipulated period, a second hospital was added in the northeastern region, totalling four maternity wards participating in the study.

From September to December 2018, nutritionists/dietitians and undergraduate students visited the maternity hospitals every weekday to identify eligible participants. All births having occurred at the time were analysed. Mothers were considered eligible when 18 years or older, having tested negative for Human Immunodeficiency Virus and Human T‐cell Lymphotropic Virus Type 1 and with singleton pregnancies. This information was collected from hospital records. For newborns, the inclusion criteria were gestational age > 37 weeks, negative for infectious diseases (that could require an extended hospital stay) and absence of congenital anomalies or neonatal conditions that could affect breastfeeding. This information was also collected from hospital records. Mothers planning to move out of the cities within 1 year were considered noneligible. After verifying the criteria, the interviewers recruited the mother–child pairs in person soon after the child was born during the ‘rooming‐in’ period. The mothers were not informed about the group to which they were assigned, as allocation status could influence their decision to accept or refuse to participate in the study.

### 2.3 Intervention Group

Counselling was offered by nutritionists/dietitians who had previously undergone training (as explained in Section 2.5). The duration of counselling was approximately 20–30 min. The major objectives were to (1) promote exclusive breastfeeding until the 6th month of age, with continued breastfeeding for 2 years, (2) guide the introduction of healthy foods beginning with the 6th month of age and (3) avoid the consumption of sugar and UPFs up to 2 years of age under the justification of protecting the period of formation of the child's food preferences.

The booklet handed to mothers and family members during the counselling session (‘10 steps to healthy eating and habits: From birth to 2 years of age’) was developed by UNICEF. The interviewers discussed (Step 2) ‘Do not offer sugar’ and (Step 8) ‘Do not offer UPFs such as sweets and soft drinks’. Additionally, family members received another colour‐illustrated booklet specially developed for the study to help them make appropriate food choices for their children. The content of both booklets is aligned with the recommendations of the ‘Dietary Guidelines for Brazilian Children Under 2 Years of Age’ issued by the Brazilian Ministry of Health [35]. Moreover, the nutritionists emphasised strategies to prolong the time of exclusive breastfeeding, such as not offering water, teas, juices, or other liquids and postponing the introduction of AS and UPFs, using the phrase ‘he/she has his/her entire life to try these foods’.

To reinforce the information provided in the maternity wards, mothers and/or family members in the intervention group also received monthly telephone calls when the children were 30 days as well as 2, 3, 4 and 5 months of age. All monthly telephone calls were conducted based on a fixed script (described in detail in Table S2). In addition to the questions, the interviewers were instructed to clarify any doubts related to infant feeding practices in accordance with the intervention booklets. For cases in which mothers or family members had questions related to other health issues, the families were advised to seek care at local primary care units of the Brazilian universal healthcare system. Upon completing 6 months, another telephone call was made to confirm the address of the families and schedule the first home visit. When telephone contact was not possible, the interviewers used the address provided by the mothers in the maternity wards.

### 2.4 Control Group

Mothers who gave birth in the weeks randomised to the control group were interviewed after enrolment and received the standard hospital routine. All BFHI‐participating hospitals offer a discharge orientation session on breastfeeding practices and appropriate referrals to ensure that mothers and infants are seen by a health worker in the first days of life. The participants were contacted again when the children were 6 and 12 months of age to schedule home visits.

### 2.5 Interviewer Training

At the beginning of each phase of the study, interviewers at all centres underwent training sessions to improve the uniformity of the data collection process. In August 2018, all teams joined online meetings carried out by the main investigator to receive detailed instructions on how to invite participants, conduct interviews, administer the intervention, and complete the questionnaires. The manual developed for the enrolment phase contained all the lines that interviewers should say at every point of the intervention.

In March and August 2019, the main investigator provided in‐person training to interviewers at each of the participating centres. At both times, a standardised 2‐day training programme was developed by the coordinating centre for use across all centres. The complete programme involved four 2–3 h sessions with a PowerPoint presentation, a manual explaining all items in the questionnaire and specific manuals for collecting data on dietary and anthropometric characteristics, serum haemoglobin and dental variables. In all sessions, the interviewers were encouraged to ask questions and clarify any doubts. After each session, practical exercises on anthropometric measurements and 24‐h recalls were conducted. Mothers and infants of the same age as the study participants were invited to these exercises so that the interviewers could rehearse the interview process before data collection. The main investigator assessed and corrected the technique when necessary. The teams also received training on how to respond appropriately to women and family members who may have questions about feeding their babies in line with the information provided during the intervention and through telephone contacts, encouraging families allocated to the intervention group to use the booklets. Regular online meetings were also held for follow‐up and to provide relevant feedback, reinforce the key points of the training as well as share experiences and lessons learned in the field.

### 2.6 Data Collection

Baseline characteristics were obtained during the first phase of data collection at the maternity hospitals soon after enrolment in the control group and before the intervention in the intervention group. A standard questionnaire with 82 questions was administered by the interviewers. Data were collected on the pregnancy (taken from patient records), maternal health, sociodemographic characteristics, and child's health since birth. In this study, we assessed characteristics of the mothers (age at childbirth, years of formal education, employment, marital status, prepregnancy and gestational conditions and monthly household income) and children (gestational age, sex, birth weight and length and type of childbirth).

Data collection at 6 and 12 months of age was performed through home visits conducted from April 2019 to January 2020. Mothers and/or family members were once again contacted by telephone by interviewers not involved in the randomisation process to schedule the interviews. In a few cases for which it was not possible to collect data at home, the participants were invited to visit other data collection sites (i.e., federal universities) and received monetary compensation to cover the costs.

The complete assessments involved the administration of questionnaires with 89 and 63 questions at 6 and 12 months of age, respectively, with both quantitative and qualitative approaches, along with haemoglobin and dental assessments at 12 months of age. The questionnaires were developed especially for the study and addressed family characteristics, infant feeding as well as mother's and child's anthropometric data. In this study, we used data obtained through the complementary food introduction questionnaires on breastfeeding, infant formula, cow's milk and toddler milk, and the consumption of AS and UPFs. In addition, we used the children's 24‐h recalls for the determination of AS and UPF consumption in grams per day collected at the two time points using the multiple‐pass method.

### 2.7 Outcomes

The randomised trial was designed to investigate eight primary outcomes, which included the consumption of AS and UPFs, weight gain, dental caries, anaemia, and breastfeeding in the first year of life. In the present study, we focused on dietary variables, with the assessment of the effectiveness of the intervention at the offer and the consumption of AS and NOVA food groups (including UPFs) at 6 and 12 months of age. The prevalence of exclusive breastfeeding up to 6 months of age, complementary breastfeeding from 6 to 12 months and the offer of infant formula, cow's milk, and toddler milk throughout the first year of life were also presented.

There are several distinctions among types of sugar (nonmilk extrinsic sugars [33], added sugars [37] and free sugars [38]). Considering that AS is the most comprehensive term and free sugars include sugars naturally present in fruit juices, widely consumed by children in Brazil, we decided to focus on AS. Examples include but are not limited to white and brown sugar, cane sugar, dextrose, molasses, and all types of syrups. Food items were considered UPFs based on the NOVA classification system, based on whether the list of ingredients contains at least one cosmetic additive or substance of rare culinary use [39]. According to NOVA, all breast milk substitutes and follow‐up formulas are classified as ultra‐processed due to the manufacturing process [38, 39]. Exclusive breastfeeding was defined as breastfeeding as the only food without the consumption of tea, water, or other liquids except for medications as well as vitamin and mineral supplements [41].

A questionnaire was created to investigate the introduction of foods to infants in the first year of life besides breast milk, such as infant formula, cow's milk, toddler milk, AS and 31 predetermined foods and beverages (15 of which are ultra‐processed). During the home visits at the 6‐ and 12‐month follow‐ups, mothers and family members were asked if the child had ever consumed the food item, in which month it was offered for the first time (introduction period) and for how long the item was offered from the 1st to the 12th month of age. The data analysed in this study considered children who had ever received AS and each of the 15 UPFs and beverages at 6 and 12 months of age and whether each infant had received breast milk and its substitutes at each month from birth to the 12th month of life.

To estimate the amount of AS and NOVA food groups (minimally processed, processed and UPFs) consumed by children in grams and calories, we assessed the 24‐h recalls collected using the multiple‐pass method at 6 and 12 months of age. During the home visits, mothers or family members were asked by a nutritionist/dietitian (trained with the same protocol) to recall and report all foods and beverages consumed in the preceding 24 h [42]. Details on food types, amounts, recipes, and brands were collected. Common household measurements (e.g., teaspoons, tablespoons, cups and serving sizes) were used to help the mothers report the amounts of food given to their children and standardise portion sizes. These measurements were subsequently converted into grams using a table for the assessment of food consumption in household measurements (Pinheiro, 2008). Breast milk consumption was not estimated in calories and was, therefore, not included in the analysis of total daily energy intake. This information was entered into the Dietbox software programme (Dietbox®, Porto Alegre, Brazil, 2013) to generate the children's food reports. All food items listed were further classified into NOVA food groups by a member of the research team not involved in the data collection process. Discrepancies were discussed with the coordinator of the study until a consensus was reached.

For the estimate of AS, each food and beverage was analysed to identify potential sources of AS. Subsequently, sugar content in food items was estimated using the food nutrition label provided by the manufacturers. When AS content was not available on the nutrition label, the Nutrition Composition Tables of Foods Consumed in Brazil [43] and the US Department of Agriculture's National Nutrient Database for Standard Reference [44] were used. If food items were not described in the tables, estimates were performed by comparisons with equivalent products. This method was based on Luie et al. [45], which has been recently adapted and validated for use in Brazil [46] and has been used by our research group in a previous publication [31].

### 2.8 Sample Size

The sample size for the study was calculated using the G*Power software package, version 3.1.9.6 (University of Düsseldorf, Düsseldorf, Germany, 2019) based on the expected reduction in the prevalence of children who received sugar at 6 months of age [30, 46]. The *z*‐test was used to estimate the difference between two independent proportions (P2 = 0.6 and P1 = 0.3), considering a power of 85% and 5% significance level. This yielded a sample size of 39 for each group. After that, a design effect of 1.3 was added, which determined a sample size of 50 children per group and 100 children per region, totalling 300 children. Considering a possible 35% dropout rate, a minimum of 462 children needed to be enroled at the beginning of the study. Post‐hoc analysis using *t* tests to estimate the difference between two independent means and *z*‐tests to estimate the difference between two independent proportions were used to calculate the achieved power of the variables used in this study, and only those with sufficient power were presented.

### 2.9 Statistical Methods

The database was managed using the Research Electronic Data Capture (REDCap) (Vanderbilt University, Tennessee, USA, 2004) hosted at the Federal University of Health Sciences of Porto Alegre. REDCap is a web‐based software platform designed to support data capture for research studies [48]. Its use enabled all collaborating centres to enter their data on the online platform, which were subsequently downloaded and analysed with the aid of the Statistical Package for the Social Sciences, version 21.0 (SPSS Statistics for Windows, IBM Corp, 2012) by the team at the coordinating centre.

The researchers who assessed the outcomes were blinded to the group to which the mother–child pairs were allocated. Generalised estimating equations (GEEs) were used to test associations and effect sizes were reported as prevalence ratios (PR) and mean difference (95% confidence interval). Prevalence ratios greater than 1 indicate a greater risk of offering AS and UPFs in the control group, whereas values less than 1 indicate a lower risk. The consumption of AS and NOVA food groups in grams were also compared through GEEs and expressed as mean and standard deviation as well as percentage of contribution to total daily energy intake. The decision to use GEEs was defined after a counselling session with a statistician not involved in the study based on the clustered and longitudinal nature of the trial, assuming the correlation of the participants included in the study [48, 49].

Sample sizes for each variable can vary according to data availability, as missing data were not imputed by the research team. The presence of outliers was investigated for quantitative variables (AS and NOVA food group intake in grams) using boxplots and no values were excluded. All data were treated as intention‐to‐treat analysis, meaning that all participants were analysed according to their original group assignment. As an additional analysis, baseline characteristics were compared between the intervention and control groups using the Mann–Whitney *U* test for continuous variables or ANOVA for categorical variables, after the determination of the distribution (normal or nonnormal) of these variables using the Kolmogorov−Smirnov test. Statistical significance was set at 5% (*p* < 0.05) for all analyses.

## 3 Results

**Figure 2:**
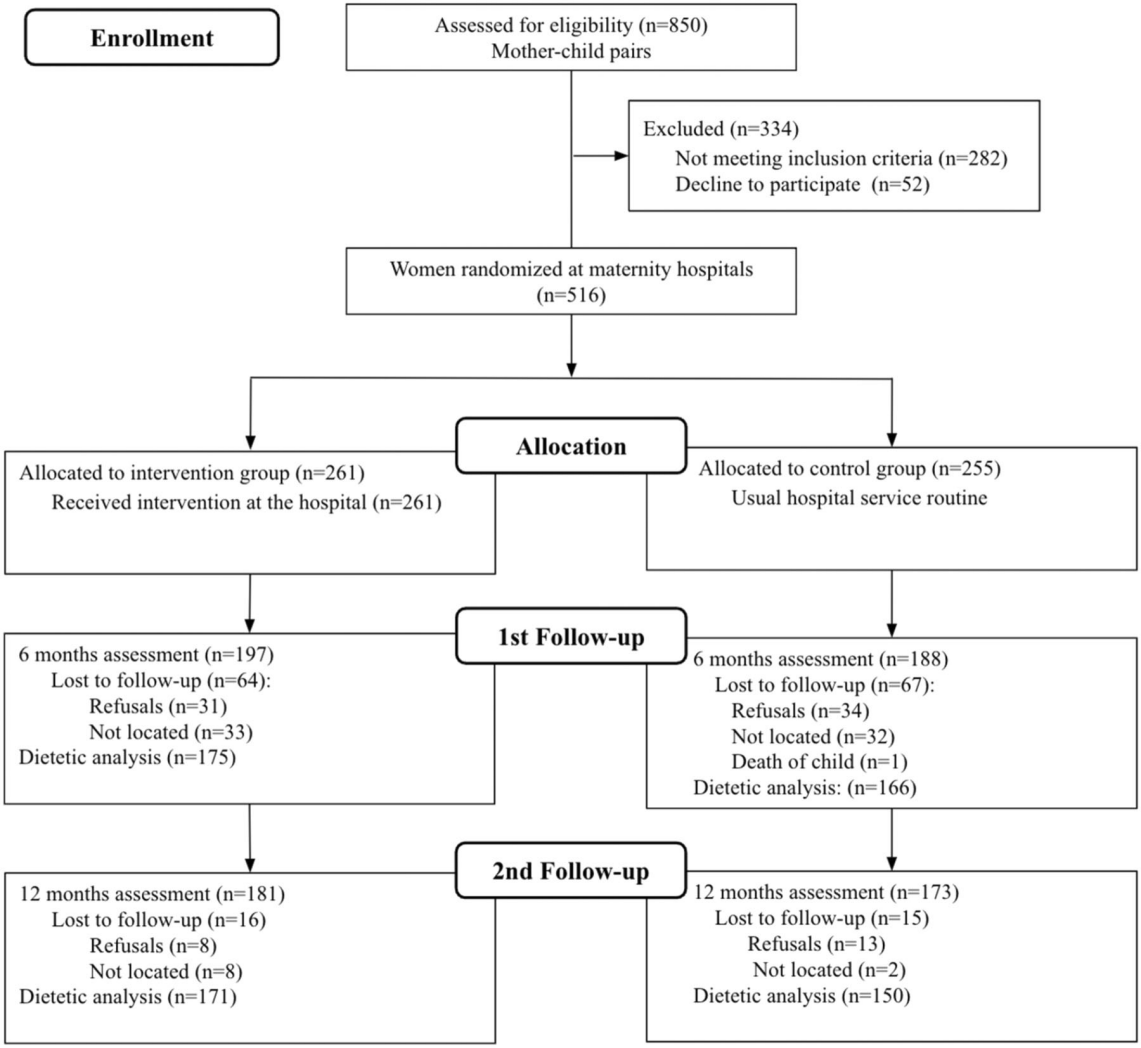
Study flow diagram.

Data collection was performed at all three centres in the same period. From September to December 2018, a total of 850 mother–child pairs were assessed for eligibility; 282 did not meet the inclusion criteria and 52 refused to participate. The major reason for not meeting the inclusion criteria was the mother's age under 18 years (144; 51.1%), gestational age less than 37 weeks (64; 22.7%) and infants with infectious diseases (55; 19.5%), such as syphilis. The 516 women who consented to participate were assigned to either the intervention (*n* = 261) or control (*n* = 255) groups according to children's week of birth. All mothers allocated to the intervention group received the study booklets during the dietary counselling session, and 149 (57.1%) received at least one of the five monthly phone calls to reinforce the intervention (*data not shown in table*). From April to August 2019, the first follow‐up was completed with 197 (75.5%) and 188 (73.7%) participants from intervention and control groups, respectively, when the children were 6 months of age. From September 2019 to January 2020, the second follow‐up was completed with 181 (69.3%) and 173 (67.8%) participants from the intervention and control groups, respectively, when the children were 12 months of age. Reasons for losses between follow‐ups are listed in Figure 2.

The baseline characteristics of the study participants are presented in Table 1. Only child's sex differed significantly between groups (males: 51.2% in the intervention group vs. 41.7% in the control group; *p* = 0.008). The mean mother's age was 27 years; 24% of mothers had ≤ 8 years of formal schooling and 43% were employed (most of them on paid maternity leave) when the interview was conducted. Pregestational BMI was ≥ 25 kg/m^2^ in nearly 50% of the sample and about 15% of families were nonnuclear. Income was lower than three times the monthly minimum wage in 77% of families (national monthly minimum wage in 2018 = R$954, equivalent to approximately 753 US dollars in 2018 currency).

**Table 1:** Baseline characteristics of participants (*n* = 516).

	Intervention (*n* = 261)^a^		Control (*n* = 255)^a^	
Characteristics	*n*/total, %		*n*/total, %	
*Mothers*				
Age at childbirth (years), *mean, SD*	27.34	6.80	27.22	6.61
Formal education, ≤ 8 years	66/260	25.4%	57/249	22.9%
Employed	111/261	42.5%	110/255	43.1%
On paid maternity leave	60/65	92.3%	65/67	97.0%
Pregestational BMI, ≥ 25 kg/m^2^	120/226	51.9%	111/219	48.1%
Gestational weight gain (kg), *mean, SD* b	12.37	7.14	11.98	6.01
First child	102/259	39.4%	102/255	40.0%
Marital status, *unmarried*	45/259	17.4%	37/255	14.5%
Household monthly income, < *3 x mmw* b	138/181	76.2%	137/178	77.0%
*Children*				
Gestational age (weeks), *mean, SD* b	39.15	1.13	39.21	1.09
Sex, *male*	147/261	51.2%	111/255	41.7%
Birth weight, < *2500 g*	6/260	2.3%	7/254	2.8%
Birth length, < *48 cm*	61/258	23.6%	73/253	28.9%
Type of childbirth, *caesarean*	103/259	39.8%	84/253	33.2%

^a^ Sample size varies according to data availability;

^b^ National monthly minimum wage in 2018 = R$954, equivalent to approximately 753 US dollars in 2018 currency.

Abbreviation: SD, standard deviation.

At 6 months of age, lower offers were found of cake (PR = 0.59; 95% CI: 0.40–0.88; *p* = 0.010), sugary milk beverages (PR = 0.82; 95% CI: 0.74–0.90; *p* < 0.001), sweetened juice (PR = 0.68; 95% CI: 0.50–0.93; *p* = 0.016) and chocolate (PR = 0.53; 95% CI: 0.29–0.97; *p* = 0.041) in the intervention group. The lower consumption of AS (PR = 0.82; 95% CI: 0.66–1.00; *p* = 0.053), cream cheese dessert (PR = 0.75; 95% CI: 0.56–1.02; *p* = 0.066) and flavoured gelatine (PR = 0.57; 95% CI: 0.33–1.01; *p* = 0.055), were considered at the threshold of statistical significance. At 12 months of age, the intervention was effective at reducing the proportion of children exposed to baby cereal (PR = 0.80; 95% CI: 0.64–1.00; *p* = 0.049), cookies with filling (PR = 0.89; 95% CI: 0.81–0.98; *p* = 0.024) and candies (PR = 0.85; 95% CI: 0.77–0.93; *p* = 0.001) (Table 2).

**Table 2:** Impact of intervention on the offer of added sugar and ultra‐processed foods was assessed using food introduction questionnaires at 6 and 12 months of age.^a^

	6 months (*n* = 385)				12 months (*n* = 354)			
Variables	Intervention	Control	PR^a^ (95% CI)	*p* value	Intervention	Control	PR^a^ (95% CI)	*p* value
	Prevalence (95% CI)	Prevalence (95% CI)			Prevalence (95% CI)	Prevalence (95% CI)		
Added sugar^a^	31.2 (28.0–34.9)	38.2 (34.8–42.0)	0.82 (0.66–1.00)	0.053	64.2 (57.9–71.1)	67.6 (61.0–74.8)	0.95 (0.77–1.16)	0.618
Ultra‐processed foods^a^								
Chocolate powder	4.7 (3.9–5.6)	5.4 (4.5–6.4)	0.86 (0.60–1.24)	0.428	20.4 (17.9–23.3)	22.9 (20.3–25.9)	0.89 (0.69–1.14)	0.365
Cake	18.3 (14.4–23.3)	30.9 (26.3–36.3)	0.59 (0.40–0.88)	0.010	75.6 (72.0–79.5)	81.9 (78.0–85.9)	0.92 (0.84–1.02)	0.112
Baby cereal	45.4 (37.8–54.6)	54.3 (46.2–64.0)	0.84 (0.59–1.18)	0.311	65.3 (57.9–73.5)	81.6 (73.6–90.4)	0.80 (0.64–1.00)	0.049
Cookies	51.2 (46.4–56.4)	55.3 (50.3–60.8)	0.92 (0.76–1.12)	0.426	87.9 (85.6–90.2)	91.0 (88.6–93.5)	0.97 (0.92–1.02)	0.200
Cookies with filling	18.6 (13.4–25.8)	19.6 (14.2–27.1)	0.94 (0.49–1.82)	0.866	36.4 (34.6–38.4)	40.9 (39.0–42.9)	0.89 (0.81–0.98)	0.024
Cream cheese dessert	43.3 (36.6–51.1)	57.4 (50.1–65.7)	0.75 (0.56–1.02)	0.066	57.5 (53.5–61.7)	64.0 (59.9–68.5)	0.90 (0.78–1.03)	0.123
Sugary milk beverage	22.1 (20.9–23.3)	27.1 (25.8–28.4)	0.82 (0.74–0.90)	< 0.001	46.2 (44.5–47.9)	47.9 (46.2–49.6)	0.96 (0.90–1.04)	0.333
Soda	5.3 (3.2–8.9)	7.8 (5.3–11.6)	0.68 (0.28–1.68)	0.407	27.0 (21.8–33.4)	33.9 (28.3–40.7)	0.79 (0.54–1.18)	0.256
Sweetened juice	5.2 (4.4–6.2)	7.6 (6.7–8.7)	0.68 (0.50–0.93)	0.016	41.3 (38.0–45.0)	41.7 (38.3–45.5)	0.99 (0.84–1.17)	0.913
Soup powder^b^	8.8 (6.1–12.8)	9.8 (6.9–14.0)	0.90 (0.43–1.85)	0.770	—	—	—	—
Chocolate	14.2 (9.7–20.7)	26.8 (21.3–33.8)	0.53 (0.29–0.97)	0.041	43.1 (36.2–51.2)	55.3 (47.8–64.0)	0.78 (0.57–1.07)	0.123
Candy	14.1 (10.4–19.1)	17.1 (13.2–22.3)	0.82 (0.47–1.45)	0.502	47.7 (45.3–50.2)	56.3 (53.7–58.9)	0.85 (0.77–0.93)	0.001
Flavoured gelatine	5.5 (3.9–7.8)	9.6 (7.7–12.1)	0.57 (0.33–1.01)	0.055	23.8 (21.3–26.6)	23.5 (20.9–26.4)	1.01 (0.80–1.27)	0.912
Ice cream	13.6 (10.5–17.5)	14.3 (11.1–18.4)	0.95 (0.57–1.57)	0.835	47.9 (42.9–53.5)	48.5 (43.3–54.3)	0.98 (0.79–1.23)	0.914
Chips^b^	6.4 (4.0–10.3)	7.7 (5.1–11.6)	0.84 (0.35–2.02)	0.696	—	—	—	—

Abbreviations: CI, confidence interval; PR, prevalence ratio.

^a^ Generalized estimating equations.

^b^ Data not collected at 12 months of age; *p* value < 0.05.

Table 3 shows the impact of the intervention on dietary outcomes measured through 24‐h recalls. The foods consumed by children were classified based on the NOVA system (Table S3). Children allocated to the intervention group had lower consumption of UPFs at 6 months of age (difference: −20.69 g/day; 95% CI: −37.87 to −3.50; *p* = 0.018) and 12 months of age (difference: −32.51 g/day; 95% CI: −61.03 to −3.99; *p* = 0.025). Lower consumption of processed foods (difference: −52.75 g/day; 95% CI: −94.36 to −11.15; *p* = 0.013) and AS (difference: −4.92 g/day; 95% CI: −9.43 to −0.41; *p* = 0.033) was also found in the intervention group at 12 months of age.

**Table 3:** Impact of intervention on consumption of NOVA food groups, added sugar and total energy assessed by 24‐h recalls collected at 6 and 12 months of age.^a^

	6 months of age				12 months of age			
	Intervention (*n* = 175)	Control (*n* = 166)	Difference (95% CI)	*p* value	Intervention (*n* = 171)	Control (*n* = 150)	Difference (95% CI)	*p* value
Variables	Mean ± SD	Mean ± SD			Mean ± SD	Mean ± SD		
NOVA food groups^b^								
Minimally processed food, g/day	209.03 ± 13.66	201.96 ± 13.67	7.06 (−30.81–44.95)	0.715	338.08 ± 19.76	321.42 ± 20.38	16.66 (−38.98–72.30)	0.557
Processed food, g/day	63.80 ± 8.43	47.34 ± 6.92	16.45 (−4.92–37.83)	0.131	100.29 ± 11.94	153.05 ± 17.55	−52.75 (−94.36 to −11.15)	0.013
Ultra‐processed food, g/day	56.69 ± 5.36	77.37 ± 6.94	−20.69 (−37.87 to −3.50)	0.018	109.97 ± 8.87	142.48 ± 11.53	−32.51 (−61.03 to −3.99)	0.025
Added sugar, g/day	7.21 ± 0.76	8.95 ± 0.92	−1.74 (−4.07– 0.59)	0.144	16.09 ± 1.40	21.01 ± 1.83	−4.92 (−9.43 to −0.41)	0.033
Total energy, kcal/day	493.81 ± 28.04	545.47 ± 31.03	−51.67 (−133.64–30.31)	0.217	844.57 ± 42.31	908.37 ± 47.85	−63.80 (−188.98–61.38)	0.318
Minimally processed food, %TE	45.35 ± 2.74	37.88 ± 2.46	7.46 (0.25–14.67)	0.043	43.89 ± 2.70	37.33 ± 2.56	6.55 (−0.73–13.83)	0.078
Processed food, %TE	11.42 ± 1.44	11.20 ± 1.46	0.22 (−3.79–4.23)	0.915	17.75 ± 2.02	18.92 ± 2.27	−1.17 (−7.13–4.78)	0.699
Ultra‐processed food, %TE	26.30 ± 2.13	34.34 ± 2.67	−8.04 (−14.73 to −1.34)	0.019	26.67 ± 2.17	29.89 ± 2.53	−3.21 (−9.75–3.32)	0.335
Added sugar, %TE	4.10 ± 0.40	5.53 ± 0.52	−1.43 (−2.72 to −0.14)	0.029	6.82 ± 0.59	8.93 ± 0.78	−2.11 (−4.04 to −0.19)	0.031

Abbreviation: %TE, percentage of total energy.

^a^ Generalized estimating equations.

^b^ Food groups described in Table S2.

**Figure 3:**
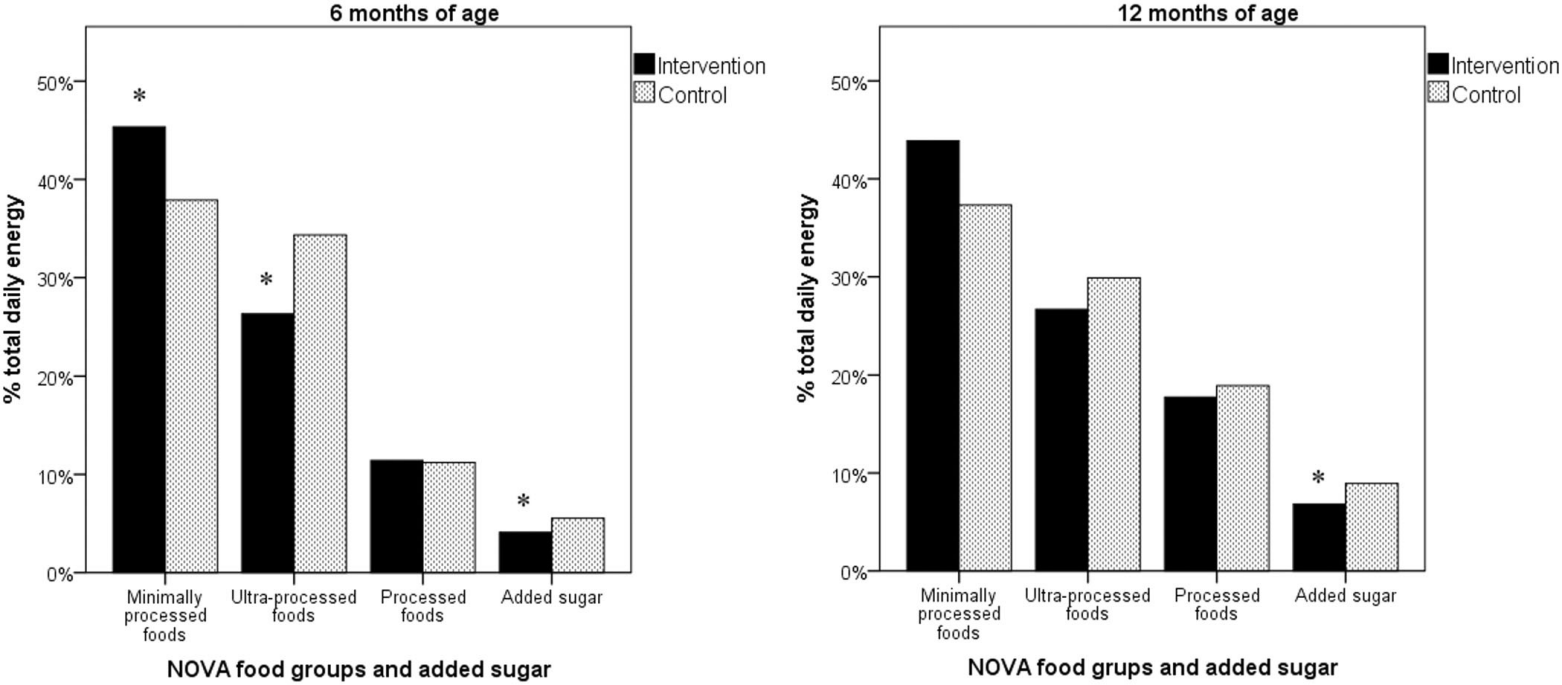
Percentage of total daily energy intake from NOVA food groups and added sugar at 6 and 12 months of age.

The percentage contribution of NOVA food groups and AS to total daily energy intake is displayed in Figure 3 and Table 3. According to the analysis, the percentage contribution of minimally processed foods to total daily energy intake at 6 months of age was higher in the intervention group than in the control group (45.35% vs. 37.88%; *p* = 0.043) and the share of UPFs (26.30% vs. 34.34%; *p* = 0.019) and AS (4.10% in intervention group vs. 5.53% in control group; *p* = 0.029) was lower in the intervention group. The intervention was effective at reducing the percentage of calories provided UPFs (6.82% in the intervention group vs. 8.93% in the control group; *p* = 0.031) to total daily energy intake at 12 months of age.

The type of milk offered to children from the 1st to the 12th month of age was investigated through food introduction questionnaires at 12 months of age. Table 4 summarises the prevalence of exclusive breastfeeding, breastfeeding, infant formula, cow's milk and toddler milk in the intervention and control groups. The intervention was effective at sustaining the offer of exclusive breastfeeding for a longer period of time, with a higher frequency in the intervention group in the first 30 days of life (92.6% vs. 88.5%; PR = 1.05; 95 CI%: 1.00−1.09; *p* = 0.046) as well as at 4 (38.2% vs. 27.2%; PR = 1.40; 95 CI% 1.20−1.64; *p* < 0.001) and 6 (14.2% vs. 6%; PR = 2.38; 95 CI: 1.75−3.25; *p* < 0.001) months of age. Fewer mothers and/or family members in the intervention group reported offering infant formula at 1 (*p* = 0.004) and 3 (*p* = 0.036) months of age, cow's milk at 4 (*p* < 0.001) months of age and toddler milk at 5 (*p* = 0.008), 8 (*p* = 0.026), 9 (*p* = 0.025) and 12 (*p* = 0.005) months of age.

**Table 4:** Impact of intervention on the offer of breast milk and substitutes from 1st to 12th month of age assessed by food introduction questionnaires at 12 months of age (*n* = 354).

	Exclusive breastfeeding^b^			Breastfeeding			Infant formula			Cow's milk			Toddler milk		
Age (months)	% IG	% CG	PR^a^ (95% CI)	% IG	% CG	PR^a^ (95% CI)	% IG	% CG	PR^a^ (95% CI)	% IG	% CG	PR^a^ (95% CI)	% IG	% CG	PR^a^ (95% CI)
< 1	92.6	88.5	1.05* (1.00–1.09)	98.2	98.2	1.00 (0.99–1.00)	6.9	10.5	0.66 (0.36–1.23)	—	—	—^c^	—	—	—^c^
1	73.9	73.7	1.00 (0.95–1.06)	96.1	93.6	1.03 (0.98–1.07)	4.1	6.3	0.64§ (0.47–0.87)	0.9	2.4	0.38 (0.85–1.74)	—	—	—^c^
2	65.1	61.8	1.05 (0.98–1.13)	95.1	92.5	1.03 (0.96–1.09)	—	—	—^c^	3.2	1.7	1.92 (0.53–6.95)	—	—	—^c^
3	52.0	44.0	1.18 (0.92–1.52)	92.0	89.1	1.03 (0.96–1.10)	12.3	16.7	0.74§ (0.55–0.98)	6.1	6.9	0.89 (0.64–1.23)	—	—	—^c^
4	38.2	27.2	1.40* (1.20–1.64)	91.6	85.1	1.08 (0.97–1.19)	11.7	16.5	0.71 (0.49–1.04)	5.6	7.3	0.76¶ (0.67–0.86)	2.8	1.4	1.98† (1.26–3.12)
5	24.1	16.8	1.44 (0.92–2.25)	88.4	81.1	1.09 (0.94–1.27)	11.6	14.3	0.81 (0.57–1.15)	9.1	12.7	0.72 (0.40–1.32)	2.4	4.0	0.60† (0.40–0.88)
6	14.2	6.0	2.38* (1.75–3.25)	87.3	80.5	1.08 (0.95–1.24)	16.5	20.3	0.81 (0.63–1.04)	20.8	21.3	0.97 (0.62–1.52)	9.7	12.5	0.77 (0.40–1.49)
7	—	—	—	83.4	76.8	1.08 (0.96–1.22)	10.5	12.0	0.87 (0.70–1.07)	18.2	19.2	0.94 (0.64–1.40)	5.1	9.3	0.55 (0.29–1.05)
8	—	—	—	82.9	75.7	1.09 (0.98–1.22)	10.0	10.6	0.94 (0.60–1.48)	19.1	21.9	0.87 (0.65–1.16)	4.4	9.6	0.46† (0.22–0.95)
9	—	—	—	82.3	72.1	1.14 (0.96–1.36)	6.4	7.5	0.85 (0.43–1.68)	17.3	23.0	0.75 (0.51–1.11)	4.3	8.4	0.51† (0.28–0.95)
10	—	—	—	81.7	71.5	1.14 (0.94–1.39)	10.4	11.3	0.92 (0.53–1.59)	23.1	29.8	0.77 (0.55–1.09)	5.8	12.1	0.48 (0.23–1.03)
11	—	—	—	79.4	69.8	1.14 (0.93–1.40)	8.8	9.1	0.96 (0.62–1.48)	20.6	22.8	0.91 (0.75–1.10)	6.2	8.9	0.69 (0.39–1.25)
12	—	—	—	77.8	67.6	1.15 (0.96–1.38)	11.1	9.3	1.20 (0.97–1.47)	39.6	46.6	0.85 (0.66–1.09)	15.5	24.8	0.62† (0.44–0.87)

* Exclusive breastfeeding at < 1 month: *p* = 0.046, 4 months: *p* < 0.001 and 6 months: *p* < 0.001.

§ Infant formula at 1 month: *p* = 0.004 and 3 months: *p* = 0.036.

¶ Cow's milk at 4 months: *p* < 0.001.

† Toddler milk at 4 months: *p* = 0.002, 5 months: *p* = 0.008, 8 months: *p* = 0.026, 9 months: *p* = 0.025 and 12 months: *p* = 0.005.

^a^ Generalized estimating equation (GEE).

^b^ Data collected at 6 months of age in intervention (*n* = 175) and control (*n* = 166) groups.

^c^ GEE criteria not met to run analysis.

## 4 Discussion

The consumption of highly processed foods rich in AS, sodium and fat increases daily energy intake and adiposity from childhood to early adulthood [50, 51]. Despite this, most intervention studies focus on the promotion of healthy infant feeding practices [52, 53] and not on the prevention of nonrecommended foods, such as UPFs. The results of our study showed that counselling mothers and family members on the health risks of AS and UPFs in a postnatal environment was effective at preventing the early introduction of UPFs and reducing the consumption of AS and UPFs in the first year of life.

With regard to reducing the early introduction of UPFs, we found a lower exposure to UPFs at 6 and 12 months of age among the children in the intervention group compared with those in the control group. A possible explanation is that family members changed their behaviour towards infant feeding practices. This may be related to the strategy employed in this study, which addressed some of the main constructs of the Health Belief Model (HBM) [55], clarifying the *severity* and *benefits* of intervention purposes during the orientation session and improving mother's sense of *barriers* and *self‐efficacy* through monthly telephone calls. When dietary educational programmes include behaviour modification techniques, the likelihood of increased efficacy is enhanced [56] due to the greater focus on specific behaviours and practices [57]. Another possible explanation for the positive results found in this study is that the UNICEF booklet contains a clear message against AS (Step 2) and UPFs (Step 8).

In addition, we found a 22.8% and 23.4% reduction in the absolute gram per day consumption of UPFs and AS at 12 months of age, respectively. This may be due to either the knowledge the mothers gained in the first 6 months, which was extended through the first year of life, and/or the lower offer of nonrecommended foods at 6 months in the intervention group helped protect the development of children's food preferences, subsequently reducing their consumption at 12 months of age. This rationale is based on the theory of food preference development, which begins in utero [58] and is reinforced by the type of milk (i.e., breast milk and/or infant formula) [59] as well as foods and beverages offered during complementary feeding [59, 60]. Previous studies showed that the early introduction of sweet‐tasting foods may promote an increase in sweet and salty taste preferences [61, 62] and affect food preferences in childhood [20, 63] even when exposure to these foods is considered minimal [65].

With regard to the contribution of NOVA food groups to total daily energy intake, the intervention provided an increase in minimally processed foods as well as a decrease in UPFs and AS at 6 months of age. However, UPF consumption accounted for nearly 30% of daily energy intake among 12‐month‐old children in both intervention and control groups, which is consistent with findings reported in studies conducted in Brazil (30%) and other low‐ to middle‐income countries, such as Mexico (29.8%) and Chile (28.6%) [64, 65, 66, 67]. The decrease in the consumption of UPFs and AS found in our study are promising results, as it is well established that early UPF consumption is associated with obesity‐related indicators in childhood [11, 12, 68].

For breastfeeding, women in the intervention group also reported a higher frequency of exclusive breastfeeding at 4 and 6 months of age compared with the control group. It is important to note that the frequency of breastfeeding in the control group at 6 months of age (6.0%) was higher than what was reported in a national study in Brazil in which only 1.3% of children are exclusively breastfed in the same age group [70]. The higher frequency of exclusive breastfeeding in the control group may also be explained by the fact that all hospitals participating in the study belong to the BFHI, which is a worldwide initiative with recognised positive impacts on breastfeeding in the short, medium, and long terms [35]. Given that neither of the two booklets focused on specific messages with regard to breastfeeding, except for Step 1 of the UNICEF booklet (*Only breast milk for the first 6 months*), it is possible that, at least in part, the emphasis on preventing nonrecommended foods enabled mothers in the intervention group to breastfeed exclusively for a longer period of time, preventing the culturally encouraged early offer of tea, breast milk substitutes, juices and homemade porridges [71]. This hypothesis is supported by evidence that breastfeeding is a long‐term protective factor for UPF consumption [71, 72, 73] and that UPF consumption is associated with the absence of breastfeeding between 6 and 24 months of age [75].

Analysing the consumption of breast milk substitutes, the effectiveness of the intervention varied with the age of the child, reflecting a current practice among low‐income families in Brazil. We found three different kinds of milk offered to infants in the first year of life: formula, cow's milk and toddler milk. The choice for each may be related to the prescription, price, informal counselling and local marketing. However, the lower prevalence of toddler milk from 5 to 12 months in the intervention group can be interpreted as one of the impacts of the intervention, as this product has AS and is classified as a UPF. In an attempt to limit the dissemination of such practices, the Brazilian Consumer Protection Institute has launched national campaigns to draw attention to toddler milk consumption [76] and the latest Lancet series on breastfeeding outlined the most common strategies used by the breast milk substitute industry [77].

To fully appreciate our results, it is important to discuss the limitations of our study. First, despite randomisation, the sex of the children was unbalanced at baseline. In this age group, however, we believe that no potential biological differences influence taste preferences and/or food consumption that could bias the results. Second, reinforcement of the intervention through telephone calls was relatively low, as not all women could be reached by telephone due to logistic issues. However, all family members allocated to the intervention group received the same booklets, which contained all information reinforced through the calls. Lastly, a single 24‐h recall with the multiple‐pass method may not be sufficient to describe an individual's usual food intake. Despite these limitations, the strengths of our study, primarily that it was a randomised, multisite study with high‐quality control, provide confidence in the results presented.

## 5 Conclusion

In conclusion, an intervention provided to mothers and family members was effective at preventing the introduction and reducing the consumption of UPFs and AS in the first year of life. Furthermore, the effectiveness of this intervention helps refute the argument that it is unrealistic to advise newborn mothers to avoid the early introduction of UPFs and AS. Thus, we believe that similar approaches could be implemented as public policies tailored to other hospitals belonging or not to the BFHI. Future studies should examine facilitators and barriers to the adherence of interventions aimed at preventing the consumption of UPFs and AS as well as the impact of these strategies on health outcomes throughout childhood.

## Author Contributions

Paola S. Baratto collected the data, performed the statistical analysis, wrote the paper, and assumed primary responsibility for the final content. Daniel J. Hoffman and Carlos A. Feldens revised the paper. Márcia R. Vitolo designed the study and revised the paper. Júlia L. Valmórbida and Paula S. Leffa collected the data and revised the paper. All authors reviewed and commented on subsequent drafts of the manuscript.

## Conflicts of Interest

The authors declare no conflicts of interest.

### 1 Peer Review

The peer review history for this article is available at https://www.webofscience.com/api/gateway/wos/peer-review/10.1111/jhn.70022.

### 2 Transparency Declaration

The lead author affirms that this manuscript is an honest, accurate, and transparent account of the study being reported. The reporting of this work is compliant with CONSORT guidelines. The lead author affirms that no important aspects of the study have been omitted and that any discrepancies from the study as planned (Clinicaltrials.gov NCT03841123) have been explained.

## Supporting information

Supporting information.

## Data Availability

Data that support the findings of this study are available from the corresponding author upon reasonable request.
